# Vaginal Bleeding in an Infant with Extreme Prematurity

**DOI:** 10.1155/2020/8881634

**Published:** 2020-08-01

**Authors:** Wing Yiu Sarah Poon, Joanna Yuet Ling Tung

**Affiliations:** ^1^Department of Paediatrics and Adolescent Medicine, Queen Mary Hospital, The University of Hong Kong, NCB 115, 102 Pokfulam Rd, Pok Fu Lam, Hong Kong; ^2^Department of Paediatrics, Hong Kong Children's Hospital, 1 Shing Cheong Road, Kowloon Bay, Kowloon, Hong Kong

## Abstract

**Background:**

Minipuberty of infancy refers to the transient activation of the hypothalamic-pituitary-gonadal (HPG) axis during the first few months of life. Studies have documented a more exaggerated and prolonged gonadotropin surge in preterm infants compared with term infants. We present a case of minipuberty presenting with vaginal bleeding at the corrected age of 3 months of life. *Case Presentation*. A former 23 + 6-week infant presented with intermittent vaginal bleeding in the diaper at the corrected age of 3 months. Physical exam showed bilateral breast buds of 0.5 cm–1 cm with no signs of pubarche. Investigations showed pubertal levels of luteinizing hormone (LH), follicle-stimulating hormone (FSH), and estradiol. As she was impressed to have exaggerated minipuberty due to extreme prematurity, no intervention was given. Repeated hormonal workup at the corrected age of 8 months showed decreasing trend of gonadotropin and estradiol levels. Vaginal bleeding resolved, and breast buds also regressed clinically.

**Conclusion:**

Our case illustrated that the robust surge of gonadotropin in an ex-premature infant can in fact result in endometrial maturation and present as vaginal bleeding. Though the mechanism of this alteration in the HPG axis in prematurity is not clearly understood, pediatricians should be aware of the benign and self-limiting nature of this phenomenon and avoid unnecessary intervention.

## 1. Introduction

Minipuberty of infancy refers to the transient activation of the hypothalamic-pituitary-gonadal (HPG) axis during the first 3–6 months of life. The rise in gonadotropin and sex steroid levels in both sexes allows maturation of sexual organs [1]. Studies have documented a stronger and more prolonged gonadotropin surge in preterm compared with term females, suggestive of an alteration in pituitary-ovarian function in this group of infants [2]. Here, we report a case where the elevated estradiol (E2) level results in endometrial maturation and shedding.

## 2. Case Presentation

A former 23 + 6-week infant, born with a birth weight of 540 grams, presented with intermittent fresh vaginal spotting on the diaper for 2-3 days at the corrected age of 3 months. Her neonatal history was significant for bronchopulmonary dysplasia (BPD), patent ductus arteriosus, retinopathy of prematurity, and grade 1 intraventricular haemorrhage which resolved on repeated ultrasound scan. As treatment for BPD, she was given inhaled fluticasone for 42 days until the corrected age of 36 weeks. She was on formula feeding with no medications except diuretics for BPD and vitamin supplement. Physical exam showed bilateral breast buds of 0.5 cm–1 cm with no other signs of puberty. Vaginal mucosa was not estrogenized, and there was no evidence of external trauma or foreign body. There was no growth spurt, and she was growing along the <3rd centile. Systemic exam was otherwise unremarkable, and there were no cafe-au-lait spots. Investigations showed luteinizing hormone (LH) 3.7 IU/L, follicle-stimulating hormone (FSH) 18 IU/L, and estradiol (E2) 167 pmol/L. Cortisol and thyroid functions were normal. Prolactin, alpha-fetoprotein, and beta-human chorionic gonadotropin levels were unremarkable. Vulval swab yielded commensals only. Ultrasound pelvis showed a pear-shaped uterus with a uterus : cervix ratio of 1.7 and a smooth endometrial echo measuring 0.14 cm in thickness, suggestive of hormonally stimulated uterus. There was no intrauterine mass or abnormal adnexal mass. Vaginal bleeding spontaneously subsided in 4 days. The gonadotropin-releasing hormone (LHRH) test performed at the corrected age of 4 months showed a predominant FSH response with a rise of LH/FSH (IU/L) from 0.49/4.3 to 16/27 at 20 mins and 13/28 at 60 mins. Magnetic resonance imaging of the brain and pituitary was unremarkable except the finding of a Rathke cleft cyst. As she was impressed to have exaggerated minipuberty due to extreme prematurity, no intervention was given. Hormonal workup at the corrected age of 8 months showed a decreasing trend of gonadotropin and estradiol levels. Breast buds regressed clinically, and there was no recurrence of vaginal bleeding. Repeated laboratory evaluation at the corrected age of 12 months showed a prepubertal value of LH < 0.1 IU/L and E2 <18.4 pmol/L. Ultrasound pelvis also revealed more tubular uterus with a fundus to cervical ratio of 1. She was last seen at the corrected age of 13 months, and there was no recurrence of pubertal development.

## 3. Discussion

Vaginal bleeding during minipuberty is reported only in a few case reports in the literature (Table 1). Similar to the reported cases, our patient belongs to the extreme prematurity group and presented during the first 6 months of life. The postnatal gonadotropin surge in these infants resembles that of central precocious puberty and results in gonadal steroidogenesis and clinical symptoms.

Onset of puberty is preceded by 2 periods of temporary HPG axis activation in early life. The first happens during midgestation and the second happens during the first 3–6 months of life, known as minipuberty. With a fall in circulating placental hormones, gonadotropin levels begin rising between days 6 and 10 after birth. Though females have lower peak LH values compared to boys, the FSH rise is more marked and sustained, and the level remain elevated until 3-4 years of age [3]. This brief period of postnatal pituitary activation is an important phase in reproductive development in both sexes. In boys, it is associated with testicular testosterone secretion, penile and testicular growth, and an increase in the numbers of Sertoli and germ cells [4]. In girls, positive association have been demonstrated between elevated E2 levels and an increase in mammary gland size and uterine growth [5]. Postnatal HPG axis activation also occurs in preterm infants and is even stronger and more prolonged. In preterm female infants, studies have shown that peak FSH and LH levels are more marked and prolonged compared to term infants, and the measurement of E2 levels in 3-month-old girls was also higher [6, 7]. Moreover, there is a stronger association between the postnatal E2 surge and the growth of the mammary gland diameter and uterine length, as well as an increase in folliculogenesis after the FSH surge [2, 5]. In our case, the initial LH and FSH levels represented central activation of the HPG axis, and she also demonstrated a prolonged and significant rise in the E2 level. As in other reported cases, the more intensive E2 surge results in breast growth and vaginal bleeding as a result of estrogenic effect on the endometrium. The condition is self-limiting, and clinical resolution is expected with a fall in gonadotropin and E2 levels.

Other causes of vaginal bleeding due to foreign body, infection, trauma, and even nonaccidental injury have to be considered in our case, and it is important to exclude these through physical examination, laboratory evaluation, and imaging. Our patient had no features of external trauma, and vulval swab was unremarkable. McCune–Albright syndrome (MAS), characterized by café-au-lait spots, gonadotropin-independent precocious puberty, various endocrinopathies, and fibrous dysplasia, should also be considered in any prepubertal female presenting with recurrent menstrual bleed. With hormonal profile suggesting central activation of the HPG axis, as well as absence of ovarian cysts on ultrasound, our patient is unlikely to have MAS. The spontaneous resolution of symptoms and that hormonal profile returned to the prepubertal range with time allowed us to establish a diagnosis of exaggerated minipuberty retrospectively.

## 4. Conclusion

Pseudomenstruation shortly after birth is well known to parents and clinicians. On the other hand, vaginal bleeding beyond the neonatal period is an alarming symptom and should be evaluated carefully. Our case illustrated that the robust surge of gonadotropin in an ex-premature infant can result in endometrial maturation and present as vaginal bleeding. Treatment with luteinizing hormone-releasing hormone (LHRH) analogs is not necessary, and clinical resolution is expected with a gradual return of gonadotropin levels to the prepubertal range. As we encounter more and more survivors of extreme prematurity nowadays, we should be aware of this phenomenon and the proper management with a conservative approach.

## Figures and Tables

**Table 1:** Summary on reported cases of preterm female infants presenting with uterine bleeding.

Author (year)	Gestation (weeks)	Corrected age on presentation	Clinical symptoms	LH (IU/L)	FSH (IU/L)	Estradiol (pmol/L)	Management and outcome

Aafke et al. (2013)	25	5 weeks	Vaginal bleeding, breast buds	6.9	4.5	964	Treated with GnRH analog for 15 months, no recurrence of pubertal development after cessation of treatment
Maria et al. (2016)	25	2.25 months	Vaginal bleeding, breast buds, facial acne, right ovarian cyst	2	5.7	253	Conservative, breast buds decreased in size, and LH and FSH returned to prepubertal levels at 5.5 mo corrected
Maria et al. (2016)	24	2.5 months	Vaginal bleeding, breast buds, facial acne, left ovarian cyst	11	5.7	451	Conservative, breast buds decreased in size, and LH and FSH returned to prepubertal levels at 6.5 mo corrected
Gisselle et al. (2017)	24	3.5 months	Vaginal bleeding, breast buds, estrogenized vaginal mucosa, and enlarged labia minora	1.3	3.1	458	Treated with GnRH analog, outcome not stated in the article

## Data Availability

No data were used to support this study.

## Abbreviations

HPG: Hypothalamic-pituitary-gonadal
LHRH: Gonadotrophin-releasing hormone
LH: Luteinizing hormone
FSH: Follicle-stimulating hormone
E2: Estradiol
BPD: Bronchopulmonary dysplasia
MAS: McCune–Albright syndrome.
